# Alkyl Thiourea Functionalised Silica for the Effective Removal of Heavy Metals from Acanthopanax senticosus Extract

**DOI:** 10.1155/2020/9860425

**Published:** 2020-03-24

**Authors:** Hong-Li Guo, Shuo Zhang, Christopher North, Min Zhang, Xiao-xia Meng, Xiu-Li Gao

**Affiliations:** ^1^State Key Laboratory of Functions and Applications of Medicinal Plants & School of Pharmacy, Guizhou Medical University, Guiyang 550025, China; ^2^College of Traditional Chinese Medicine and Lab of Traditional Chinese Medicine, Chongqing Medical University, Chongqing 400016, China; ^3^Guizhou Medical University Experimental Animal Center, Guiyang 550025, China; ^4^PhosphonicS Ltd., 114 Milton Park, Abingdon, Oxfordshire OX14 4SA, UK; ^5^Microbiology and Biochemical Pharmaceutical Engineering Research Center of Guizhou Province College and University, Guizhou Medical University, Guiyang 550004, China

## Abstract

Acanthopanax senticosus extract with excessive standard of Pb, Cd, Hg, and Cu was used as the research object, and the alkyl thiourea functionalised silica was used as a new heavy metal removal scavenger. The heavy metal removal process was optimised by orthogonal experiment with dynamic and static adsorption modes. Meanwhile, the content of Acanthopanax B and Acanthopanax E, the solid content, and the HPLC fingerprint similarity were used as quality monitoring indicators of Acanthopanax senticosus heavy metal removal before and after. Then, the technical adaptability of heavy metal removal by alkyl thiourea functionalised silica was evaluated. Under the optimal dynamic adsorption conditions, the average removal rates of Pb, Cd, Hg, and Cu were 91.64%, 93.04%, 81.77%, and 83.11%, respectively. Under the optimal static adsorption conditions, the average removal rates of Pb, Cd, Hg, and Cu were 82.22%, 89.95%, 81.26%, and 82.97%, respectively. During Acanthopanax senticosus extract heavy metal removal before and after, the change percentage of Acanthopanax B and Acanthopanax E was less than 2.00%, the solid content loss rate was only 0.18%, and the fingerprint similarity was over 99.9%. The method can be used to satisfy the high efficiency of selective removal of harmful elements in Acanthopanax senticosus extract and the effective composition of almost no effect; the method is simple and easy, so it can be recommended for pretreatment of heavy metals in Traditional Chinese Medicine extracts, and this way provides a new thought and research technique to decrease the contents of heavy metals.

## 1. Introduction

Heavy metals have obvious harmful effects on the body's metabolism and normal physiological functions. Excessive levels of heavy metals in the human body can lead to various diseases, such as diseases of the nervous system and blood system and even cancer [1, 2]. The content of heavy metals in Traditional Chinese Medicine is one of the important indicators to measure the quality of Traditional Chinese Medicine [3]. Due to the excessive pollution of heavy metals in Traditional Chinese Medicine, many incidents occurred in developed countries in Europe and the United States and many were detained which has seriously restricted its international development level [4] and has a great negative impact on the international reputation of Traditional Chinese Medicine; hence, it has attracted the attention of the government and medical science and technologist [5, 6]. In order to solve the current drug safety and the emergency situation facing export, to overcome the problem of Traditional Chinese Medicine contaminated by harmful elements, it is necessary to find a rapid, convenient, and suitable method for large-scale removal of harmful elements in Traditional Chinese Medicine [7], to strengthen the development and application of various removal technologies and adsorption materials, and to remove the harmful element units in the production of Traditional Chinese Medicine, such as extraction, to control the harmful metal content of the finished medicine at a reasonable level [8]. Due to the complexity of the form of harmful metals in Traditional Chinese Medicine, such as complexation or embedding with effective active ingredients and background values, traditional methods for removing harmful metals in recent years, such as alcohol precipitation, molecular imprinting, activated carbon adsorption, ion-exchange resin, and superfluid methods, still have many defects such as low yield, weak adsorption, large dosage, high cost, and loss of active ingredients [9, 10]. At present, it is only in the experimental research stage and is only reported in the literature, but it still lacks the industrial production mode [11]. Therefore, it is urgent to develop new harmful metal removal technologies with high selectivity, strong adsorption, and good industrial application value, which at the same time can protect the amount and efficacy of active ingredients [12].

Alkyl thiourea functionalised silica is a multifunctional polymer-modified silica material with a highly specific coordination group [13]. Currently, the removal of toxic catalysts in the world's synthetic pharmaceutical industry and the recovery of precious metals in the petroleum industry have been extensively applied [14–16], but the application of harmful element removal in Traditional Chinese Medicine extracts has not been reported in domestic and foreign literature. According to literature reports, silica gel loses its adsorption property after it absorbs water by 17%, which is only a carrier (macroporous resin method and activated carbon are difficult to meet this requirement), and the hydroxyl group on the surface can be coordinated by a modified heteroatom containing S and N (can be strongly complexed with heavy metal specificity) [17]. It can be predicted that the kinds of new solid adsorption technology will have broad market prospects in the field of removal of heavy metals in Traditional Chinese Medicine.

Acanthopanax senticosus is a very important Traditional Chinese Medicine. It has the same effect as ginseng. It can regulate the body's function, improve the body, and enhance the body's immunity, especially in the aspects of antifatigue, improving sleep, antioxidation, and filling gas blood, which all have very good effect to the body's health [18]. This study and the British PhosphonicS Ltd. company jointly carried out the use of alkyl thiourea functionalised silica as a model adsorption material, with Acanthopanax senticosus extract as a heavy metal pollution research object, to investigate the removal effect of Pb, Cd, Hg, and Cu; meanwhile, the content of Acanthopanax B and Acanthopanax E, the solid content, and the HPLC fingerprint similarity were used as quality monitoring indicators of Acanthopanax senticosus heavy metal removal before and after. Our study is aimed at judging the adaptability of alkyl thiourea functionalised silica to remove heavy metals under the premise of protecting the active ingredients of Traditional Chinese Medicine. Furthermore, it provides a reference for the feasibility evaluation of this type of material and it resolves the drug safety and international development problems caused by excessive heavy metals in Traditional Chinese Medicine.

## 2. Materials and Methods

### 2.1. Materials

The materials used were as follows: Cd/Cu/Pb/Hg standard stock solution (National Nonferrous Metals and Electronic Materials Analysis and Testing Center); ICP-MS tuning fluid (Thermo Ltd.); ICP-MS Mass Calibration Solution (Thermo Ltd.); nitric acid (Suzhou Crystal Rui Chemical Co., Ltd.); hydrogen peroxide (Chongqing Chuanjiang Chemical Reagent Factory); alkyl thiourea functionalised silica (PhosphonicS Ltd., UK); Acanthopanax senticosus extract (Shaanxi Tiandiyuan Biotechnology Co., Ltd.); and Acanthopanax B and Acanthopanax E (Guizhou Dida Biotechnology Co., Ltd.).

### 2.2. Methods

#### 2.2.1. Preparation of Acanthopanax senticosus Extract Solution

Weigh 50 g of Acanthopanax senticosus extract in a 200 mL volumetric flask; add about 150 mL of ultrapure water; add 2 mL of Pb, Cd, Hg, and Cu; mix standard solution with a concentration of 100 *μ*g·mL^−1^; perform ultrasound for 15 minutes at room temperature; and then make up to 200 mL with ultrapure water.

#### 2.2.2. Quantitative Analysis of Pb, Cd, Cu, and Hg

Precisely measure 2.0 mL of Acanthopanax senticosus solution adsorbed before and after. Add 6 mL of HNO_3_ (GR) and 2 mL of H_2_O_2_ (GR) in a Teflon bottle, shake it and mix it, seal, and predissolve for 10 hours. Digest by microwave digestion instrument, cool to room temperature, volatilize the acid to 1~2 mL liquid at 120°C, transfer it to a 25 mL volumetric flask, dilute to volume with ultrapure water, and determine Pb, Cd, Hg, and Cu simultaneously by ICP-MS.

#### 2.2.3. Optimization of Static Adsorption Method and Condition

Weigh the appropriate amount of alkyl thiourea functionalised silica in a round bottom flask, add the Acanthopanax senticosus solution (Section 2.2.1), and put it into the air bath constant temperature oscillator to oscillate. The amount of alkyl thiourea functionalised silica, the oscillation frequency, the oscillation temperature, and the shock time were as a single factor investigated in this study, to determine the optimal range of each factor. On this basis, the orthogonal test of 4 factors and 3 levels was designed; the overall rating of average removal rates of heavy metals was used as the process evaluation index; then, the optimal static adsorption conditions of removal of heavy metals in the Acanthopanax senticosus was determined. The method for determination of heavy metals in optimal static process was referred in Section 2.2.2.

*(1) Effect of Alkyl Thiourea Functionalised Silica Dosage.* The amount of medicinal material/adsorbed dose was 30, 50, 80, and 120, respectively. The adsorption conditions (25°C, 260 times·min^−1^, and 600 min) were used to analyze the effect of the amount of adsorbent on the removal rate.

*(2) Effect of Oscillation Frequency.* The amount of controlled medicinal material/adsorbed dose was 80, at low speed (80 times·min^−1^), medium speed (160 times·min^−1^), high speed (260 times·min^−1^), and super high speed (360 times·min^−1^) frequencies. Heavy metals in Acanthopanax senticosus solution were adsorbed for 600 min at 25°C. The effect of oscillation frequency on the removal rate was analyzed.

*(3) Effect of Adsorption Temperature.* The amount of controlled medicinal material/adsorbed dose was 80, and the heavy metals in the Acanthopanax senticosus solution were adsorbed for 600 min at a temperature of 15, 25, 35, and 45°C and 260 times·min^−1^, respectively. The effect of absorption temperature on the removal rate was analyzed.

*(4) Effect of Adsorption Time.* The amount of controlled medicinal material/adsorbed dose was 80, 25°C and 260 times·min^−1^, and the heavy metals in Acanthopanax senticosus solution were adsorbed for 10, 30, 60, 120, 200, 400, 600, and 800 min, respectively. The effect of absorption time on the removal rate was analyzed.

*(5) Orthogonal Test Design.* In order to investigate the interaction of various factors on the removal rate of heavy metals, based on the single-factor experiment, the orthogonal combination test of L9(34) was designed based on four factors: adsorption dosage, adsorption time, oscillation frequency, and adsorption temperature. The residual amount of Pb, Cd, Cu, and Hg in the Acanthopanax senticosus solution was determined (refer to Section 2.2.2).

#### 2.2.4. Dynamic Adsorption Method and Condition Optimization

Accurately weigh the alkyl thiourea functionalised silica with the medicinal material/adsorbed dose which was 80, wetly load the column, equilibrate with ultrapure water, collect the liquid solution after passing through the solid adsorption column, and measure the residual amount of Pb, Cd, Cu, and Hg in the sample solution. In order to discuss the optimal dynamic adsorption process, this study evaluated the effects of different 4 factors of aspect ratio, elution speed, temperature, and sample loading on the removal rate. On this basis, the orthogonal test of 4 factors and 3 levels was designed to remove the heavy metals. Comprehensive scores of average removal rates of heavy metals were used as the process evaluation index to determine the optimal dynamic adsorption conditions of removal of heavy metals in the Acanthopanax senticosus. The method for determination of heavy metals in optimal dynamic process was referred in Section 2.2.2.

*(1) Influence of the Aspect Ratio.* Select three different adsorption columns (the diameter to height ratios are 1 : 10, 1 : 15, and 1 : 20); the loading is 100 mL, the elution temperature is 25°C, and the elution rate was 5 BV·h^−1^ (the elution rate of a BV is equivalent to the volume of a solid adsorption column), and the effect of the aspect ratio on the removal rate was analyzed.

*(2) Effect of the Elution Rate.* Select a column with an aspect ratio of 1 : 20; the loading was 100 mL, the elution temperature was 25°C, and the elution rate was 3, 5, 8, and 10 BV·h^−1^, respectively. The effect on the removal rate was analyzed.

*(3) Effect of Sample Loading.* Select a column with diameter to column height of 1 : 20. The elution temperature is 25°C, the elution rate is 3 BV·h^−1^, and the sample loading is 100, 200, 300, and 500 mL, respectively. The effect on the removal rate was analyzed.

*(4) Effect of Elution Temperature.* Select a column with an aspect ratio of 1 : 20, elute at 3 BV·h^−1^, select 100 mL for sample loading, and analyze the temperature for removal at temperatures of 15, 25, 35, and 45°C, respectively. The effect on the removal rate was analyzed.

*(5) Orthogonal Test Design.* In order to investigate the interaction of various factors on the removal rate of heavy metals, based on the single-factor experiment, this paper will design the orthogonal combination of L9(34) with four factors: aspect ratio, sample loading, elution speed, and temperature. Measure 900 mL of Acanthopanax senticosus solution (Section 2.2.1); divide it into 9 parts, each 100 mL of liquid medicine; test according to the orthogonal design method; and determine residual content of Pb, Cd, Cu, and Hg in each sample (refer to Section 2.2.2).

#### 2.2.5. Quality Evaluation of Acanthopanax senticosus Heavy Metal Removal Before and After

The content of Acanthopanax B and Acanthopanax E, the solid content, and the HPLC fingerprint similarity were used as quality monitoring indicators of Acanthopanax senticosus heavy metal removal before and after. Then, the technical adaptability of heavy metal removal by alkyl thiourea functionalised silica was evaluated.

*(1) Determination of Acanthopanax B and Acanthopanax E in Liquid Medicine.*

1. Preparation of the Test Solution. Precisely measure 1 mL of Acanthopanax solution (25% by mass) where heavy metals were removed before and after to 20 mL volumetric flask, make up to 80% methanol solution (volume), and perform ultrasound for 25 min; a test solution of about 12.5 mg·mL^−1^ was obtained, the test solution was filtered by 0.22 *μ*m microporous membrane, and then the filtrate was collected for analysis
2. Preparation of the Reference Substance Acanthopanax B Solution. Accurately weigh 0.48 mg of Acanthopanax B, make up to 5 mL (80% methanol solution) (mass concentration 96 *μ*g·mL^−1^), and perform ultrasound for 5 min
3. Preparation of the Reference Substance Acanthopanax E Solution. Accurately weigh 0.51 mg of Acanthopanax E, make up to 5 mL (80% methanol solution) (mass concentration 102 *μ*g·mL^−1^), and perform ultrasound for 5 min
4. Preparation of the Mixed Reference Solution of Acanthopanax B and Acanthopanax E. Accurately weigh 0.53 mg and 0.60 mg of reference substance Acanthopanax B and reference substance Acanthopanax E, make up to 5 mL (80% methanol solution) (concentrations of Acanthopanax B and Acanthopanax E were 106 *μ*g·mL^−1^ and 120 *μ*g·mL^−1^, respectively), and perform ultrasound for 5 min

*(2) Analysis of the Solid Content.* Taking the Acanthopanax senticosus heavy metal removal solution before and after as the research object, weigh 25 mL into the evaporating dish which had been dried to constant weight and evaporate to dryness in a boiling water bath. The drying was continued in a blast-drying oven at 105°C. Weigh it and calculate the solid content and loss rate of the liquid.

*(3) HPLC Fingerprint Similarity Evaluation.* To prepare the test solution, precisely measure 2 mL of the Acanthopanax heavy metal removal solution (25% by mass) before and after to a 10 mL volumetric flask, dilute to the mark with ultrapure water, and perform ultrasound for 25 min. The concentration of test solution was 50 mg·mL^−1^. Filter with 0.22 *μ*m microporous membrane and collect the continuous filtrate for analysis.

#### 2.2.6. Principle of Heavy Metal Removal by the New Kinds of Scavenger

**Figure 1:**
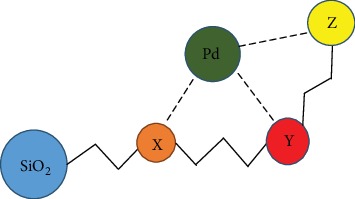
Schematic diagram of heavy metal removal.

Figure 1 shows that heavy metal (Pd) is present in the Traditional Chinese Medicine freely or chemically bonded to the active ingredient in Traditional Chinese Medicine (e.g., Y or Z). The alkyl thiourea-bonded silica (SiO_2_ bonding organic functional group X) used in this study is competitively heavy metal-binding with the active ingredient of the Traditional Chinese Medicine (e.g., Y or Z), thereby performing heavy metal removal, similar to the tug-of-war effect. Hence, the alkyl thiourea-bonded silica forms a chelate with the heavy metal.

## 3. Results

### 3.1. Static Adsorption and Condition Optimization Results

#### 3.1.1. Effect of the Amount of Scavenger on the Removal Rate of Heavy Metals

**Figure 2:**
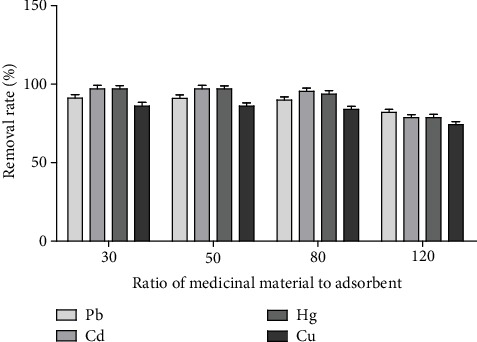
Effect of the amount of scavenger on the removal rate of heavy metals (*n* = 3).

Figure 2 shows that the removal rate of Pb, Cd, Hg, and Cu was close to the maximum when the ratio of medicinal material/adsorbent dose was 80. If the ratio of medicinal material/adsorbent dosage continues to increase, the removal rate of heavy metals was significantly reduced.

#### 3.1.2. Influence of Oscillation Frequency on the Removal Rate of Heavy Metals

**Figure 3:**
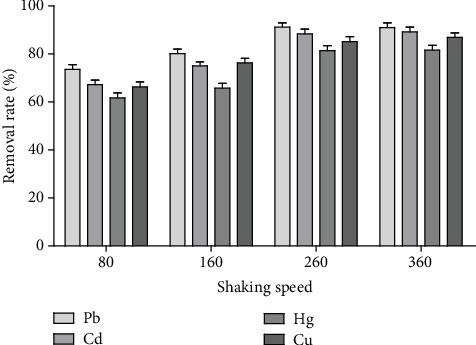
The effect of shaking speed on the removal rate of heavy metals (*n* = 3).

Figure 3 shows that within a certain oscillation frequency range, the removal rate of heavy metals was proportional to the oscillation frequency. When the oscillation frequency reached 260 times·min^−1^, the removal rate of heavy metals no longer increased significantly.

#### 3.1.3. Effect of Adsorption Temperature on the Removal Rate of Heavy Metals

**Figure 4:**
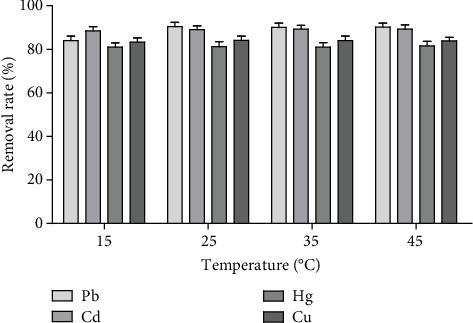
The effect of temperature on the removal rate of heavy metals (*n* = 3).

Figure 4 shows that the effect of temperature on the removal rate of heavy metals was not obvious.

#### 3.1.4. Effect of Adsorption Time on the Removal Rate of Heavy Metals

**Figure 5:**
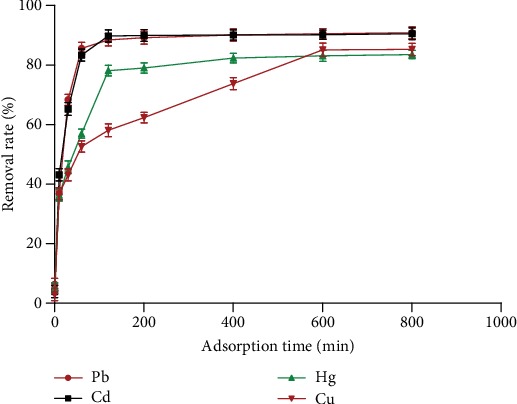
The effect of scavenging time on the removal rate of heavy metals (*n* = 3).

Figure 5 shows that the adsorption time was proportional to the removal rate in a certain adsorption time. The removal rates of Pb, Cd, and Hg did not increase significantly after 120 min of adsorption time, but the removal rate of Cu did not increase after 600 min. Pb, Cd, and Hg had a faster removal rate than Cu since the adsorption reaches a short saturation time.

#### 3.1.5. Orthogonal Test Analysis Results

From the results of the orthogonal test (Table 1), it can be seen that the influence of four factors on the removal rate of heavy metals was significant. The primary and secondary order was B>C>A>D. The D factor with the smallest difference was used as the error term for variance analysis. The analysis results (Table 2) indicated that factor B had a significant difference, which indicated that the adsorption time has a greater impact on the removal rate of heavy metals. According to the analysis results, the optimal static adsorption process conditions for Pb, Cd, Hg, and Cu in the aqueous solution of Acanthopanax senticosus extract were A_1_B_3_C_3_D_3_.

#### 3.1.6. Verification Test Results for Optimal Process Conditions

The following optimal adsorption process conditions were adopted: the amount of medicinal material/adsorbent was 80, the oscillation frequency was 260 times·min^−1^, the adsorption time was 600 min, the adsorption temperature was 45°C, and the 6 parallel tests were carried out to verify the process conditions and obtain higher removal.  Table 3 shows that the removal rate of heavy metals was more than 81% and the RSD value was all less than 2.00, so this process was reasonable and reliable.

### 3.2. Dynamic Adsorption and Condition Optimization Results

#### 3.2.1. Effect of Diameter to Column Height on the Removal Rate of Heavy Metals

**Figure 6:**
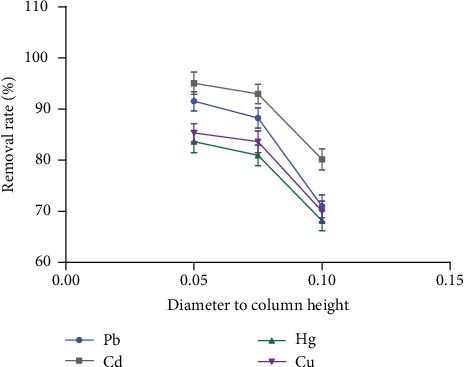
The effect of diameter to column height on the removal rate of heavy metals (*n* = 3).

Figure 6 shows that the removal rate of Pb, Cd, Hg, and Cu was the largest when the effect of diameter to column height was 0.05. The smaller the diameter to column height, the higher the column efficiency and the higher the removal rate of heavy metals, but the greater the resistance and the slower the flow rate. Therefore, the choice of the diameter to column height depends on the viscosity of the sample to be purified.

#### 3.2.2. Effect of the Elution Rate on the Removal Rate of Heavy Metals

**Figure 7:**
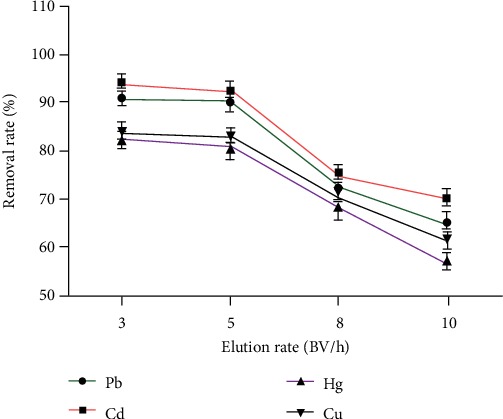
The effect of washing speed on the removal rate of heavy metals (*n* = 3).

Figure 7 shows that in the dynamic adsorption process, the elution rate was directly related to the removal rate of heavy metals. The faster the elution rate, the worse the removal effect was. When the elution rate reached 5 BV·h^−1^, the purpose of efficiently removing heavy metals can be satisfied.

#### 3.2.3. Effect of Sample Loading on the Removal Rate of Heavy Metals

**Figure 8:**
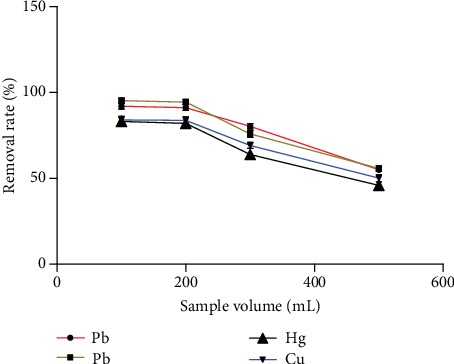
The effect of sample volume on the removal rate of heavy metals (*n* = 3).

Figure 8 shows that there was a direct relationship between the amount of sample and the removal rate of heavy metals. The results showed that the removal rate of heavy metals was close to the maximum when the sample volume was 200 mL.

#### 3.2.4. Effect of Elution Temperature on the Removal Rate of Heavy Metals

**Figure 9:**
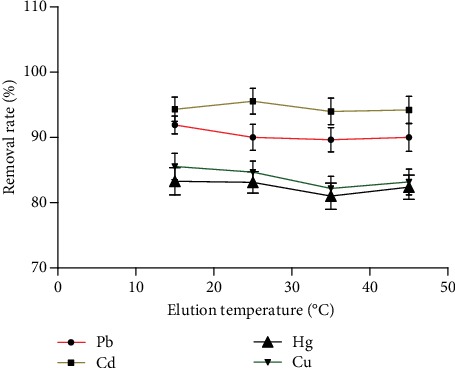
The effect of washing temperature on the removal rate of heavy metals (*n* = 3).

Figure 9 shows that the effect of temperature on the removal rate of heavy metals was not obvious.

#### 3.2.5. Orthogonal Test Analysis Results

From the results of the orthogonal test (see Table 4), it can be seen that the influence of four factors on the removal rate of heavy metals was significant. The primary and secondary order was F>G>E>K, and the K factor with the smallest difference was used as the error term for variance analysis. The results of the analysis of variance (Table 5) indicated that both factors F and G have significant differences and indicated that both the loading and elution rates had a large effect on the removal rate of heavy metals. According to the analysis results, the optimal dynamic adsorption process conditions for removal of Pb, Cd, Hg, and Cu in the aqueous solution of Acanthopanax senticosus extract were E_3_F_1_G_1_K_1_.

#### 3.2.6. Verification Test Results for Optimal Process Conditions

The optimal adsorption process conditions were as follows: the diameter to column height ratio was 1 : 20, the sample loading was 100 mL, the elution rate was 3 BV·h^−1^, the elution temperature was 15°C, and the 6 parallel tests were performed to verify the process conditions and obtain higher. The metal removal rate and the RSD value were both less than 2.00%. The results are shown in Table 6. So the process of metals removed was reasonable and reliable.

### 3.3. Result Quality of Acanthopanax senticosus Heavy Metal Removal Before and After

#### 3.3.1. The Content of Acanthopanax B and Acanthopanax E in Sample Liquid

Table 7 shows that active ingredients Acanthopanax glycosides B and E were not affected by heavy metal removal before and after, and the rate of change was less than 2%.

**Figure 10:**
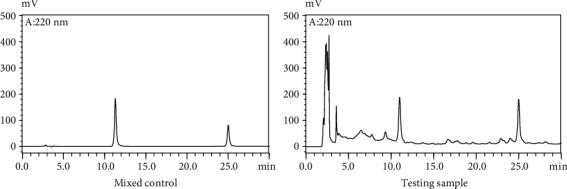
HPLC chromatogram.

Figure 10 displays the mixed control and testing sample HPLC chromatogram.

#### 3.3.2. Analysis of Solid Content of the Drug Solution Adsorption Before and After

The solid content of the liquid before and after removal was 227.38 mg·mL^−1^ and 225.62 mg·mL^−1^, respectively, and the loss rate was only 0.18%, which indicated that there was no obvious loss of solid content before and after removal of heavy metals by using new solid adsorbent.

#### 3.3.3. Evaluation Results of HPLC Fingerprint Similarity Adsorption Before and After

**Figure 11:**
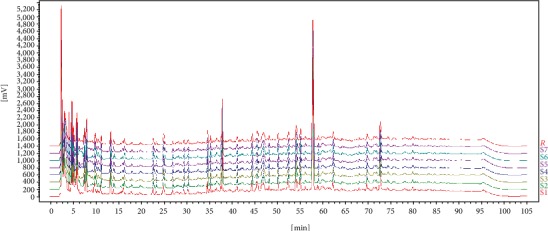
Evaluate results of the similarity on fingerprint with or without metal removal.

The HPLC fingerprints of Acanthopanax senticosus heavy metal adsorption before and after were analyzed. The results showed that the active composition ingredients of Acanthopanax senticosus did not change. The “Traditional Chinese Medicine fingerprint similarity evaluation system (version 2.0)” issued by the National Pharmacopoeia Committee was adopted, which analyzed Acanthopanax senticosus after heavy metal removal and obtained the control fingerprint by using the preadsorption spectrum as a reference. Based on this, the overall similarity evaluation was performed, and 6 parallel experiments were performed. The chromatographic peaks of the HPLC spectra before and after removal of heavy metals were matched with each other, and the similarity evaluation results were all greater than 99.9%. The results are shown in Figure 11.

## 4. Conclusion

In this study, the samples of the extracts of Traditional Chinese Medicine to be purified that exceeded the standard of heavy metals were prepared, and the main production operation units involved in the extraction process of Traditional Chinese Medicine, such as stirring extraction and dynamic column, were designed. The static stirring mode and dynamic column mode were used to remove heavy metals. The process research, while examining the single-factor influence removal rate and orthogonal experiment optimization optimal process, obtained a better removal effect; dynamic and static adsorption can achieve more than 80% heavy metal removal rate.

The carrier of the novel solid adsorption bonding material used in the research is silica gel. When the water content of silica gel exceeds 17%, the adsorption property is not suitable. It is especially suitable for Chinese medicine liquid (aqueous liquid or alcohol liquid); that is, the adsorbent is harmful in removing heavy metals. At the same time, the elements do not significantly affect the active ingredients of Traditional Chinese Medicine. By comprehensively analyzing the content of index components, solid content loss rate, and HPLC fingerprint before and after removing heavy metals from Acanthopanax senticosus extract aqueous solution, it is further verified that the alkyl thiourea-bonded silica gel removed Pb, Cd, Hg, and Cu. The chemical composition has not changed significantly, and the experimental design purpose of selective and high-efficiency removal of heavy metals under the premise of protecting the active constituents of Acanthopanax senticosus extract (unilateral medicinal materials) has been achieved, and it has good industrial application value.

## Figures and Tables

**Table 1:** The results of the orthogonal test.

Serial number	A	B	C	D	Results (rate removal of heavy metals (%))				
					Pb	Cd	Hg	Cu	Average rate of removal
1	1	1	1	1	65.66	58.69	43.97	45.97	53.57
2	1	2	2	2	85.63	89.93	81.79	61.69	79.76
3	1	3	3	3	87.37	91.12	82.27	84.97	86.43
4	2	1	2	3	66.12	63.00	49.76	43.03	55.48
5	2	2	3	1	82.19	87.27	76.25	65.37	77.76
6	2	3	1	2	72.34	65.34	59.33	63.14	65.04
7	3	1	3	2	66.31	63.00	46.28	41.26	54.21
8	3	2	1	3	69.37	65.08	62.00	58.08	63.63
9	3	3	2	1	80.06	73.17	63.07	74.25	72.64
K1	73.25	54.42	60.75	67.99					
K2	66.09	73.72	69.23	66.37					
K3	63.49	74.70	72.80	68.51					
*R*	9.76	20.28	12.05	2.18					

**Table 2:** The results of analysis of variance.

Source of variance	SS	*f*	MS	*F*	*P*
A	153.28	2	19.79	19.00	<0.05
B	784.75	2	101.32	19.00	<0.05
C	230.63	2	29.78	19.00	<0.05
D (errors)	7.75	2	1.00	19.00	

Note: *P* < 0.05 indicates that the difference is statistically significant.

**Table 3:** The test results of the optimal process conditions.

Sample serial number	Rate removal of heavy metals (%)			
	Pb	Cd	Hg	Cu
1	83.31	89.26	82.89	85.17
2	83.29	91.47	83.03	83.12
3	84.27	90.12	80.28	82.26
4	85.34	90.91	81.79	84.53
5	83.27	89.25	82.51	82.64
6	83.43	91.33	80.01	83.39
Average	82.12	89.21	81.27	82.47
RSD	1.27	1.49	1.78	1.34

**Table 4:** The results of the orthogonal test.

Serial number	E	F	G	K	Results (rate removal of heavy metals (%))				
					Pb	Cd	Hg	Cu	Average rate of removal
1	1	1	1	1	82.07	85.13	82.31	85.95	83.89
2	1	2	2	2	80.16	79.55	78.00	79.71	79.36
3	1	3	3	3	60.07	63.08	67.33	64.93	63.85
4	2	1	2	3	85.13	89.61	72.14	73.05	80.11
5	2	2	3	1	79.39	82.18	68.66	72.37	75.65
6	2	3	1	2	79.60	73.33	61.76	67.34	70.51
7	3	1	3	2	79.88	85.06	75.64	77.08	79.42
8	3	2	1	3	92.67	95.05	82.17	84.96	88.72
9	3	3	2	1	78.69	84.38	69.12	71.65	75.96
K1	75.70	81.13	81.03	78.42					
K2	75.88	81.09	78.43	76.76					
K3	80.78	70.19	72.89	77.18					
*R*	5.08	10.91	8.15	1.65					

**Table 5:** The results of analysis of variance.

Source of variance	SS	*f*	MS	*F*	*P*
E	49.85	2	11.237	19.00	
F	237.40	2	53.52	19.00	<0.05
G	103.87	2	23.45	19.00	<0.05
K (errors)	4.46	2	1.00	19.00	

Note: *P* < 0.05 indicated that the difference is statistically significant.

**Table 6:** The test results of the optimal process conditions.

Sample serial number	Rate removal of heavy metals (%)			
	Pb	Cd	Hg	Cu
1	91.10	93.57	80.11	85.21
2	90.21	95.17	82.19	81.47
3	93.17	92.67	83.57	84.15
4	91.24	92.33	80.37	83.64
5	92.16	91.27	82.87	80.78
6	91.76	93.01	81.22	83.17
Average	91.67	93.00	81.76	83.19
RSD	1.26	1.39	1.97	1.99

**Table 7:** Determination results of Eleutheroside B and Eleutheroside E with or without metal removal.

Indicator components	Quality score (%)							Rate of change (%)
	Control	Sample 1	Sample 2	Sample 3	Sample 4	Sample 5	Sample 6	
Acanthopanax B	0.556	0.561	0.563	0.559	0.547	0.566	0.567	1.35
Acanthopanax E	0.957	0.960	0.982	0.969	0.972	0.971	0.978	1.67

## Data Availability

The data sets used and/or analyzed during the current study are available from the corresponding author on reasonable request.
